# QuickStats

**Published:** 2013-12-06

**Authors:** Sally C. Curtin

**Figure:**
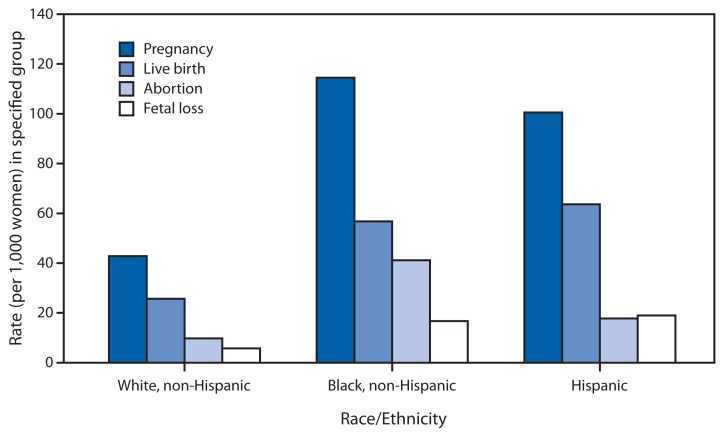
Rates of Pregnancies and Pregnancy Outcomes Among Teens Aged 15–19 Years, by Race/Ethnicity — United States, 2009

The pregnancy rate for non-Hispanic white teenagers aged 15–19 years (42.8 per 1,000) was less than half that of non-Hispanic black (114.5) and Hispanic teenagers (100.5). Hispanic teenagers aged 15–19 had the highest birth rate of all groups (63.6 per 1,000), whereas non-Hispanic black teenagers had the highest abortion rate (41.1 per 1,000). Fetal loss rates were more than twice as high for non-Hispanic black (16.7 per 1,000) and Hispanic teenagers (19.0) than for non-Hispanic white teenagers (7.3).

**Source:** Curtin SC, Abma JC, Ventura SJ, Henshaw SK. Pregnancy rates for U.S. women continue to drop. NCHS data brief no. 136. Hyattsville, MD: US Department of Health and Human Services, National Center for Health Statistics, CDC; 2013 (in press).

